# Population structure of Purple Sandpipers (*Calidris maritima*) as revealed by mitochondrial DNA and microsatellites

**DOI:** 10.1002/ece3.2927

**Published:** 2017-03-31

**Authors:** Nathalie M. LeBlanc, Donald T. Stewart, Snaebjörn Pálsson, Mark F. Elderkin, Glen Mittelhauser, Stephen Mockford, Julie Paquet, Gregory J. Robertson, Ron W. Summers, Lindsay Tudor, Mark L. Mallory

**Affiliations:** ^1^ Department of BiologyAcadia UniversityWolfvilleNSCanada; ^2^ Department of Life and Environmental SciencesUniversity of IcelandReykjavíkIceland; ^3^ Department of Natural ResourcesGovernment of Nova ScotiaKentvilleNSCanada; ^4^ Maine Natural History ObservatoryGouldsboroMEUSA; ^5^ Canadian Wildlife Service, Environment and Climate Change CanadaSackvilleNBCanada; ^6^ Wildlife Research Division, Environment and Climate Change CanadaMount PearlNLCanada; ^7^ Lismore, 7 Mill CrescentNorth KessockRoss‐shireUK; ^8^ Maine Department of Inland Fisheries and WildlifeBangorMEUSA

**Keywords:** *Calidris maritima*, conservation genetics, microsatellites, migration, mtDNA, phylogeography, Purple Sandpipers

## Abstract

The Purple Sandpiper (*Calidris maritima*) is a medium‐sized shorebird that breeds in the Arctic and winters along northern Atlantic coastlines. Migration routes and affiliations between breeding grounds and wintering grounds are incompletely understood. Some populations appear to be declining, and future management policies for this species will benefit from understanding their migration patterns. This study used two mitochondrial DNA markers and 10 microsatellite loci to analyze current population structure and historical demographic trends. Samples were obtained from breeding locations in Nunavut (Canada), Iceland, and Svalbard (Norway) and from wintering locations along the coast of Maine (USA), Nova Scotia, New Brunswick, and Newfoundland (Canada), and Scotland (UK). Mitochondrial haplotypes displayed low genetic diversity, and a shallow phylogeny indicating recent divergence. With the exception of the two Canadian breeding populations from Nunavut, there was significant genetic differentiation among samples from all breeding locations; however, none of the breeding populations was a monophyletic group. We also found differentiation between both Iceland and Svalbard breeding populations and North American wintering populations. This pattern of divergence is consistent with a previously proposed migratory pathway between Canadian breeding locations and wintering grounds in the United Kingdom, but argues against migration between breeding grounds in Iceland and Svalbard and wintering grounds in North America. Breeding birds from Svalbard also showed a genetic signature intermediate between Canadian breeders and Icelandic breeders. Our results extend current knowledge of Purple Sandpiper population genetic structure and present new information regarding migration routes to wintering grounds in North America.

## 1 Introduction

Purple Sandpipers (*Calidris maritima*) breed from the Canadian High Arctic to southern Hudson Bay, as well as in Greenland, Iceland, mainland Norway and Russia, and northern islands from Svalbard (Norway) east to the Severnaya Islands (Russia; Cramp & Simmons, 1983). They migrate from these breeding areas to overwinter along the northeastern coast of North America, southern Greenland, Iceland, and also along the coasts of northwest Europe (Cramp & Simmons, 1983). Migratory routes between breeding and wintering sites of North American Purple Sandpipers were largely unknown until a recent study confirmed a trans‐Atlantic route from the Canadian Arctic in the summer to Scotland and Ireland in the winter (Summers et al., 2014). Wintering aggregations of Purple Sandpipers may contain birds from multiple breeding locations (Corse & Summers, 1999; Nicoll, Summers, Underhill, Brockie, & Rae, 1988). For example, the majority of Svalbard breeders migrate to the Norwegian coast and western Sweden (Hake, Blomqvist, Pierce, Järås, & Johansson, 1997), but small numbers winter in northeast Scotland (Summers et al., 2010) where they mix with individuals from Canada (Summers et al., 2014). Their extreme northern breeding and northern wintering ranges, their complex migratory patterns and their relatively recent colonization of the Arctic following the retreat of the Wisconsin glaciation, make this a fascinating species for both ecological and evolutionary studies.

**Figure 1:**
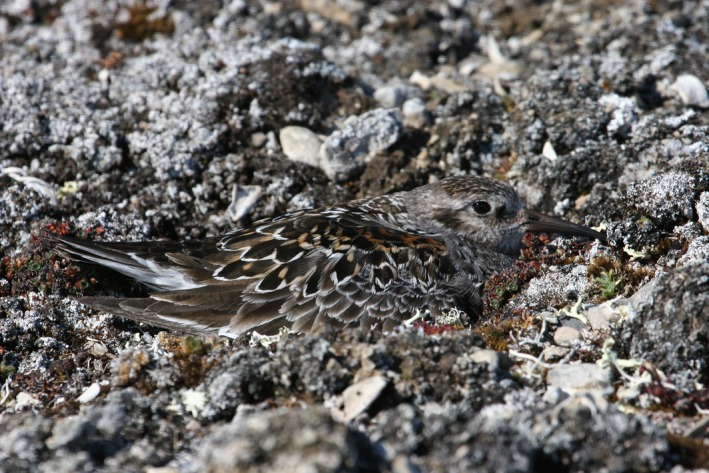
Photograph of a Purple Sandpiper, *Calidris maritima*

The genetic structure of some Purple Sandpiper populations has recently been examined by Barisas, Amouret, Hallgrímsson, Summers, and Pálsson (2015). These authors used mitochondrial (ND2 and COX1) and nuclear gene DNA sequences (a sex‐linked nuDNA intron, RANBP3L, and four autosomal nuDNA introns, HMG‐2, PDCD11, TGFβ2, RPL30) and morphometric data (wing, culmen, tail, and tarsus lengths) to assess the validity of three potential subspecies, *C. maritima maritima*,* Calidris m. littoralis*, and *Calidris m*. *belcheri* (Figure 1). These subspecies were proposed based on morphological analyses of several breeding populations conducted by Engelmoer and Roselaar (1998). Barisas et al. (2015) examined *C. m. maritima* from Canada, Svalbard (Norway), and Greenland and *C. m. littoralis* from Iceland. They concluded that although there were notable differences among breeding populations in both morphology and DNA sequence data, these differences were not large enough to warrant distinct subspecific status. Barisas et al. (2015) also suggested that the pattern of genetic differentiation was consistent with a model of recent expansion from a single refugium after the last ice advance, and because Purple Sandpipers in Svalbard generally had the highest genetic diversity, that the location of the historical refugium for this species may have been in that geographic region.

Past efforts to identify and describe migration routes of Purple Sandpipers by banding have been successful in parts of the range (e.g., West Greenland: Salomonsen, 1967; Norway: Rae, Nicoll, & Summers, 1986; Iceland: Summers, Corse, Nicoll, Smith, & Whitfield, 1988; and Svalbard: Hake et al., 1997), but less is known about the migratory patterns of Canadian populations (Mittelhauser, Tudor, & Connery, 2006; Morrison, 1984; Summers et al., 2014). Banding and resighting or telemetry for identifying migration routes have often had limited success for this species due to the difficulty in resighting and recapturing individuals (Payne & Pierce, 2002). Morphometric discrimination has also been used to infer the breeding origin of some wintering populations (Hallgrimsson, Summers, Etheridge, & Swann, 2012; Mittelhauser et al., 2006). However, the practice of using morphological differences to identify migration routes has generally been limited by the overlap in measurements among populations and by the presence of sexual size dimorphism that is of the same magnitude as that found between some populations (Barisas et al., 2015; Burton & Evans, 2001).

Molecular studies have also been conducted on other closely related Arctic scolopacids. Wenink, Baker, Rosner, and Tilanus (1996) demonstrated strong genetic structure in Dunlin (*Calidris alpina*), and Wennerberg, Marthinsen, and Lifjeld (2008) used mtDNA and microsatellites markers to determine breeding origin of wintering populations of Dunlins on a fine scale. Pruett and Winker (2005) used mitochondrial DNA to assess historical patterns of genetic differentiation in Rock Sandpipers (*Calidris ptilocnemis*) and found evidence for the use of multiple refugia across Beringia that corresponded to different glacial cycles. Rock Sandpipers, which are distributed along the Pacific coast in northwestern North America, Kamchatka, and the Aleutian Islands (Pruett & Winker, 2005), are considered to be the sister species to Purple Sandpipers, with both species occupying breeding and wintering grounds at the same latitude. Both Rock and Purple sandpipers have garnered interest because of their ability to winter in cold environments (Ruthrauff, Dekinga, Gill, Van Gils, & Piersma, 2015; Summers, Strann, Rae, & Heggås, 1990). Their similar patterns of habitat use, migratory behaviors, and close phylogenetic relatedness make them excellent subjects for evolutionary and ecological comparisons.

One behavioral characteristic that could affect the pattern of population differentiation in Purple Sandpipers is their apparent high fidelity to wintering locations (Atkinson, Summers, Nicoll, & Greenwood, 1981; Dierschke, 1998; Mittelhauser, Tudor, & Connery, 2012; Summers, Nicoll, & Peach, 2001). Behavioral fidelity to a particular breeding location can lead to population differentiation due to reduced gene flow even in a species with high dispersal potential (Avise, 2009) such as a migratory shorebird. Fidelity of adult Purple Sandpipers to breeding locations is less well known although there are a few studies that provide some insight into this behavior. For example, the return rate of adult birds in a north Scotland population was 65% (Smith & Summers, 2005), and in Svalbard, the return rate was 54% for males and 60% for females (Payne & Pierce, 2002). Natal philopatry was also measured in a small population of breeding birds in north Scotland and was 4.5% (Smith & Summers, 2005), indicating that relatively few juveniles birds returned to their natal site to breed.

In this study, we use two mitochondrial DNA fragments (portions of the Control Region and the Cytochrome b gene) and ten newly developed, polymorphic microsatellite markers to extend the genetic analysis conducted by Barisas et al. (2015). We assess genetic diversity and population structuring of Purple Sandpipers across a large portion of their global range to evaluate whether the breeding origin of migratory, wintering birds can be determined using genetic data.

## 2 Materials and Methods

### 2.1 Sample collection

Blood, tissue, and pre‐isolated DNA samples were obtained for a total of 279 Purple Sandpipers from a variety of sources, outlined in Figure 1. The 20 samples from Nunavut were dried toe pads donated by the Canadian Museum of Nature, and ages varied from 37 to 62 years old. Details for individual samples can be found in Table S3. Three blood samples taken from Rock Sandpipers were kindly provided by Dr. Dan Ruthrauff (U.S. National Park Service, Arctic Network) and DNA extracted, amplified, and sequenced from them was used as an out‐group when constructing phylogenetic trees.

All but five of the Canadian breeding samples we analyzed were obtained from areas covered in the comparison of morphological characteristics published by Engelmoer and Roselaar (1998). We placed the 10 samples collected from breeding populations on Bathurst Island, Cornwallis Island, and Prince of Wales Island in northern Nunavut in the subspecies *C. m. maritima* based on Engelmoer and Roselaar (1998). *Calidris m. maritima* is also the putative subspecies of most European breeding populations. Our five samples taken from Long Island and North Twin Island in southern Hudson Bay were placed in the putative subspecies *C. m. belcheri*, again based on the morphological patterns described by Engelmoer and Roselaar (1998). The five samples taken from Coats Island and the West Foxe Islands in northern Hudson Bay are from the border between the ranges of *C. m. maritima* and *C. m. belcheri*. Engelmoer and Roselaar (1998) did not examine the birds that breed on these islands, nor were morphological measurements available for our samples. Because we did not know a priori to which putative subspecies the birds from Coats and West Foxe islands belonged, we performed two separate sets of analyses, one in which these samples were included in *C. m. maritima* and one in which these samples were included in *C. m. belcheri*. Because there were no strong differences in interpretation between these two scenarios (detailed in LeBlanc, 2015), we present only the results obtained for the analysis in which the northern Hudson Bay Purple Sandpipers were placed in *C. m. maritima*. Hereafter, the northern *C. m. maritima* samples will be referred to as the northern Nunavut population, and the southern *C. m. belcheri* samples will be referred to as the southern Hudson Bay population.

### 2.2 Laboratory procedures

DNA was isolated from all tissue types using a DNeasy Blood and Tissue kit (Qiagen), spin column protocol. For blood samples, we used 190 μl phosphate buffered saline in step 1b of the animal blood protocol as recommended by Bush, Vinsky, Aldridge, and Paszkowski (2005). For liver samples, we cut 25 mg from the main tissue using flame sterilized utensils. For feathers, because only one feather was available per sample, samples were cut into ~1 cm pieces, soaked in a 10% bleach solution for 30 min, and then rinsed three times in distilled water to maximize DNA yield (Speller, Nicholas, & Yang, 2011). These pieces were then incubated overnight at 56°C in 180 μl buffer ATL (Qiagen), 20 μl 1 mol/L DTT, 20 μl 1 mol/L proteinase K (Taberlet & Bouvet, 1991). The final elution step was extended to 5 min before the DNA was eluted. All other steps were as described in the DNeasy kit spin column protocol.

The mitochondrial DNA control region and cytochrome b mtDNA fragments were amplified separately in reaction mixtures containing 44 μl Platinum^®^ Blue PCR SuperMix, 2 μl forward primer (10 μmol/L), 2 μl reverse primer (10 μmol/L), and 2 μl template (~25 ng/μl) or up to 5 μl for DNA from feathers, which typically had <5 ng/μl DNA concentration. When possible, the control region and cytochrome b regions were amplified in one fragment. However, for some samples, which were presumably partly degraded, the control region was amplified in three fragments and cytochrome b was amplified in two fragments. Control region reactions were heated to 94°C for 3 min for initial denaturing, followed by 40 cycles of 1 min at 94°C for denaturing, 1 min at 50°C for annealing, and 1 min at 72°C for extension. Finally, the temperature was brought to 72°C for 5 min for a final extension. Cytochrome b reactions were heated to 94°C for 3 min for denaturing, followed by 40 cycles of 45 s at 94°C for denaturing, 45 s at 50°C for annealing, 1 min at 72°C for extension, and the final extension step of 72°C for 5 min. Unpurified PCR products were sent to McGill University and Génome Québec Innovation Centre where they were purified and Sanger sequenced on an Applied Biosystem 3730xl DNA Analyzer using the same primers as in the amplification reactions.

Initial attempts to amplify an 841‐bp fragment of the mtDNA control region from all samples used primer L98 taken from Wenink, Baker, and Tilanus (1993) and primer H1018, which was designed by us using Primer3 (Untergasser et al., 2012). DNA samples that did not amplify (mostly from feathers) were re‐amplified in two or three fragments. Primers were again taken from either Wenink et al. (1993) or designed in‐lab. Rock Sandpiper DNA was amplified using an alternate primer also designed in‐lab.

When possible, a cytochrome b fragment of 712 bp was amplified using primers designed for Rock Sandpipers (Pruett & Winker, 2005). Samples that failed to amplify using these primers were amplified in two fragments using internal primers developed in‐lab. All samples were sequenced using the same primers with which they were amplified, in both directions with approximately 90% overlap, and sequence data from both directions were combined to maximize sequence length. Sequences that contained unique, singleton mutations were resequenced to confirm the haplotype.

As mitochondrial DNA is inherited as a single locus, the control region and cytochrome b sequences were concatenated and analyzed as a single marker (Ma et al., 2012). Sequences were initially aligned to the complete mitochondrial genome of Arenaria interpres (Accession number AY074885), the most closely related organism for which the entire mitochondrial genome is available. Sequences were trimmed to remove nucleotide positions with missing data, including a section of cytochrome b (*n* = 19 bp) that was not covered by the internal primers. All primers are listed in Table S1 and Figure S1.

Primers were developed to amplify 10 polymorphic microsatellite loci by Ecogenics GmbH (Schlieren, Switzerland; detailed in Table S2). Primers were designed with a universal 18 base pair M13 tail and co‐amplified with an M13 primer labeled with FAM fluorescent dye. Microsatellite loci were amplified in the following duplex combinations: CM2668 and CM2988, CM705 and CM997, CM296 and CM3007, CM1669 and CM2198. Loci CM1422 and CM3547 were amplified individually. Duplex combinations were chosen based on the following criteria: (1) fragment sizes of the two loci did not overlap; and (2) when amplified together and then run on a gel, the bands produced by both loci were of similar intensity. We added a greater concentration of primer for the “shorter” fragment in the reaction (see Table S2 for fragment sizes), following recommendations in Neff, Fu, and Gross (2000). All PCRs were run in a Biometra T‐Gradient thermocycler using the following protocol: 94°C for 15 min; 26 cycles of 95°C for 30 s, 56°C for 45 s, 72°C for 45 s; eight cycles of 95°C for 30 s, 53°C for 45 s, 72°C for 45 s; 72°C for 5 min.

All microsatellite reactions were performed in 20 μl volumes using Platinum^®^ Taq polymerase (Invitrogen, Carlsbad, CA, USA) and associated reagents (2 μl 10× Platinum^®^ Taq buffer, 0.4 μl 10 mmol/L dNTPs [0.2 mmol/L], 0.1 μl Platinum^®^ Taq, 0.8 μl 50 mmol/L MgCl_2_ [2 mmol/L], 2 μl template DNA). Individual reactions used 2 μmol/L primer concentrations in the following volumes: 0.4 μl forward primer, 1.6 μl reverse primer, 1.6 μl M13 primer, with 11.1 μl of water to bring the total volume to 20 μl. Duplex reactions used 2 μmol/L primer concentrations in the following volumes: 0.26 μl of the smaller fragment's forward primer, 0.2 μl of the larger fragment's forward primer, 1.0 μl of the smaller fragment's forward primer, 0.8 μl of the larger fragment's forward primer, 1.5 μl of the M13 primer, and 7.94 μl water to bring the volume up to 20 μl.

Because of limited quantity of DNA, the northern Nunavut samples were pre‐amplified (Arandjelovic et al., 2009; De Barba & Waits, 2010; Hedmark & Ellegren, 2006) in a 10‐μl reaction with all 10 primer pairs, using Platinum^®^ Taq polymerase and associated reagents (2 μl 10× buffer, 0.22 μl 10 mmol/L dNTPs, 0.3 μl each of 10 μmol/L forward primers, 0.3 μl each of 10 μmol/L reverse primers, 0.1 μl Taq, 0.93 μl MgCl_2_, 5 μl template DNA, and 5.75 μl water). The product was then diluted 1:100 in water, and 5 μl was used as template for 10 singleplex reactions (2 μl 10× buffer, 0.22 μl 10 mmol/L dNTPs, 0.63 μl of 2 μmol/L forward primer, 2.5 μl of 2 μmol/L reverse primer, 2.5 μl of 2 μmol/L M13 primer, 0.07 μl Taq, 0.47 μl MgCl_2_, 5 μl template DNA, and 6.61 μl water). Negative controls were included in all amplifications, and samples were amplified and genotyped three times to ensure accurate genotypes. All microsatellite PCR products were sent to McGill University and Génome Québec Innovation Centre, where they were genotyped using an ABI 3730xl DNA Analyzer.

### 2.3 Mitochondrial DNA data analysis

Sequences were aligned using Clustal Omega (Sievers et al., 2011), edited in Jalview 2.8 (Waterhouse, Procter, Martin, Clamp, & Barton, 2009), and saved in FASTA format. Cytochrome b sequences were translated into amino acid sequences in MEGA 6 (Tamura, Stecher, Peterson, Filipski, & Kumar, 2013) to check for premature stop codons, frameshifts, or other evidence of nuclear pseudogenes (Rodríguez, Albornoz, & Domínguez, 2007). FaBox 1.41 (Villesen, 2007) was used to concatenate sequences and convert the fasta files into the format required for Arlequin 3.5.1.3 (Excoffier & Lischer, 2010) and TCS 1.21 (Clement, Posada, & Crandall, 2000). PGDSpider 2.0.8.0 (Lischer & Excoffier, 2012) was used to convert between input formats for all other software described.

We ran jModeltest 2.1.4 (Darriba, Taboada, Doallo, & Posada, 2012; Guindon & Gascuel, 2003) on the control region and cytochrome b sequences separately. The best model for the sequences combined and for the control region alone was the HKY+I model. One of the two best models for cytochrome b was the HKY model. In the interests of simplifying the analysis, and because there were so few variable sites for the cytochrome b sequence, the HKY+I model was used to construct the Maximum Likelihood (MLB) and Bayesian phylogenies on the concatenated data set.

### 2.4 Microsatellite DNA data analysis

The presence of null alleles was tested for using ML‐NullFreq (Kalinowski & Taper, 2006) and Micro‐checker v. 2.2.3 (Van Oosterhout, Hutchinson, Wills, & Shipley, 2004), which look for departures from Hardy–Weinberg equilibrium due to heterozygote deficit. A comparison of null allele detection programs carried out by Dąbrowski et al. (2014) found that these analyses have a high probability of giving false positives. This study also found that the detection of false positives tends to be inconsistent between programs and data sets (Dąbrowski et al., 2014). By recognizing only the presence of null alleles that were detected by both ML‐NullFreq and Micro‐checker, the probability of false positives was decreased considerably while still retaining power to detect true null alleles (Dąbrowski et al., 2014). We tested for the presence of null alleles in all populations, with the knowledge that mixed wintering locations are expected to show deviations from Hardy–Weinberg proportions (Dharmarajan, Beatty, & Rhodes, 2013). Finally, pairwise linkage disequilibrium values were calculated using FSTAT 2.9.3.2 (Goudet, 1995). Due to the large number of linkage disequilibrium tests performed, results were analyzed accounting for multiple tests two ways. Following the recommendation of Waples (2015), we looked at overall number of positives compared to our nominal Type I error rate (5%) and assessed whether the overall number of positive results was greater or less than “expected” number of false positives. We also analyzed results using a sequential Bonferroni correction (Rice, 1989).

### 2.5 Genetic diversity

Standard genetic diversity indices of mtDNA sequences were calculated in DnaSP 5.10.1 (Librado & Rozas, 2009) for each population as well as for all populations pooled. Indices included number of haplotypes, number of private haplotypes (i.e., those occurring only in one population), number of segregating sites, nucleotide diversity (average pairwise nucleotide difference between individuals, Nei, 1987), and haplotype diversity (probability of any two individuals randomly selected from the population having different haplotypes, Nei, 1987). Observed and expected heterozygosities of microsatellite loci were calculated in Arlequin, and allelic richness was calculated using FSTAT to determine relative diversity levels of all populations. Private allele richness was calculated using HP Rare v. June‐6‐2006 (Kalinowski, 2005), again for microsatellite loci.

### 2.6 Population structure (Φ_ST_ and *F*_ST_)

Overall genetic differentiation among breeding populations and pairwise differentiation among all populations was calculated in Arlequin 3.5.1.3 (Excoffier & Lischer, 2010) using Φ_ST_ for mtDNA data and *F*_ST_ for microsatellite data. Wintering populations, which contain a mixture of birds from several breeding populations, were included in pairwise estimates because similarity between a wintering population to a breeding population may provide information on migration routes. Due to patterns seen in STRUCTURE clustering results for microsatellite loci, the four samples from western Newfoundland (Broom Point) were treated as a separate wintering location for pairwise estimates. *p*‐Values to evaluate statistical significance were generated using 10,000 nonparametric permutations to obtain a null distribution of pairwise Φ_ST_ values. Estimated number of migrants between populations was calculated using Arlequin.

### 2.7 Phylogenetic analyses

Three phylogenetic trees were constructed in total using mtDNA haplotype data. Two were made using MEGA 6 (Tamura et al., 2011), and a third was made using the program Mr Bayes 3.2.2 (Ronquist & Huelsenbeck, 2003). The two trees constructed in MEGA 6 included a MLB tree, made using the HKY+I model of nucleotide substitution, pairwise deletion, and the Nearest‐Neighbor‐Interchange Heuristic Method; and a maximum parsimony tree (MPB) made using the Subtree‐Pruning‐Regrafting search method and pairwise deletion. Both trees were bootstrapped 1,000 times. Mr Bayes was used to construct a tree using a Bayesian algorithm, using the HKY+I model of nucleotide substitution. Two simultaneous analyses were run to calculate convergence values. For both analyses, a run length of 1,000,000 and a burn‐in fraction of 25% were used. Uncorrected *p*‐distances were calculated for major clades found, using MEGA 6. A statistical parsimony haplotype network was constructed in TCS 1.21 (Clement et al., 2000) for all mtDNA fragments combined as well as for just the protein‐coding cytochrome b fragments.

### 2.8 STRUCTURE clustering

A Bayesian clustering analysis was performed using STRUCTURE 2.3.3 (Pritchard, Stephens, & Donnelly, 2000). This analysis was performed on all samples without defining putative populations. Analyses were carried out using the admixture method for *K* = 1–5, where *K* is the putative number of clusters, and run for 2,000,000 cycles with a burn‐in of 200,000 cycles. The most likely number of genetic clusters was determined using the method described in Evanno, Regnaut, and Goudet (2005). Because this analysis did not use defined populations, results for wintering individuals are shown using more localized areas, particularly for Maine samples.

### 2.9 Demographic history

We used DnaSP to measure departures in our sequence data from a neutral model of evolution using Tajima's *D*, Ramos‐Onsin's *R* ^2^, Fu's *F*s and Mismatch Distribution (Rogers & Harpending, 1992; Slatkin & Hudson, 1991). The program BOTTLENECK 1.2.02 (Piry, Luikart, & Cornuet, 1999) was used to test for the evidence of a recent population bottleneck (Cornuet & Luikart, 1996). Analyses used three different models: (1) a strict stepwise mutational model; (2) the infinite alleles model; and (3) a more complex two‐phase model that assumes mutation via both the stepwise and the infinite alleles model (Di Rienzo et al., 1994). Studies of avian microsatellite data have found that typically 60%–80% of mutations are acquired via a stepwise mutation model (Miller, Haig, Mullins, Popper, & Green, 2012), and so the two‐phase model was implemented using each end of this range as a parameter. We specified two‐phase model variances as 4, 9, 16, 25 or 36, according to the observed allele sizes across all loci (Di Rienzo et al., 1994). Results over all loci were analyzed with the Wilcoxon Signed‐Rank test (Cornuet & Luikart, 1996).

## 3 Results

### 3.1 Genetic diversity

Of the 1,535 nucleotide sites in the control region and cytochrome b sequenced from a total of 279 birds, 41 sites were polymorphic, 22 of which occurred in the control region and 19 in cytochrome b (Table 1). A total of 42 haplotypes was obtained, 23 of which were shared by at least two individuals. Of these 42 haplotypes, 22 haplotypes were only found in wintering locations, and nine were found only in breeding locations. The most common haplotype comprised 33% of samples and was found in all breeding and wintering locations. The second most common haplotype comprised 17% of samples and was found in all wintering locations but only in one breeding location (southern Hudson Bay).

**Table 1:** Standard diversity estimates for breeding and wintering populations of Purple Sandpipers (*Calidris maritima*), as well as all samples pooled together

	mtDNA						Microsatellites					
	*N*	*H*	Pr	*S*	π	*ĥ*	*A*_T_	*P*_T_	*A*_R_	*P*_R_	*H*_O_	*H*_E_
Iceland	18	4	2	4	0.00063 (0.00014)	0.660 (0.078)	4.6	0.20	3.38	0.26	0.60 (0.18)	0.62 (0.19)
Svalbard	20	9	5	9	0.00081 (0.00016)	0.789 (0.086)	5.6	0.10	3.92	0.31	0.66 (0.18)	0.68 (0.15)
N. Nunavut	15	8	2	11	0.00133 (0.00038)	0.733 (0.124)	4.6	0.00	3.35	0.12	0.57 (0.17)	0.61 (0.12)
S. Nunavut	5	3	0	5	0.00169 (0.00043)	0.800 (0.164)	3.3	0.00	3.30	0.06	0.54 (0.23)	0.60 (0.16)
Breeding total	58	20	–	20	0.00102 (0.00014)	0.782 (0.049)	4.5	0.08	3.49	0.19	0.59	0.63
Maine	115	19	5	21	0.00156 (0.00008)	0.797 (0.025)	6.8	0.60	3.34	0.13	0.58 (0.15)	0.60 (0.12)
New Brunswick	21	11	0	13	0.00187 (0.00016)	0.914 (0.038)	5.0	0.00	3.35	0.12	0.58 (0.10)	0.61 (0.11)
Nova Scotia	28	13	0	17	0.00160 (0.00023)	0.812 (0.072)	5.1	0.10	3.39	0.14	0.56 (0.14)	0.60 (0.13)
Newfoundland	16	10	2	11	0.00173 (0.00028)	0.905 (0.054)	4.6	0.20	3.47	0.17	0.60 (0.18)	0.63 (0.13)
Scotland	42	23	7	24	0.00170 (0.00020)	0.920 (0.032)	5.8	0.20	3.68	0.22	0.58 (0.24)	0.64 (0.14)
Wintering Total	221	33	–	33	0.00167 (0.00007)	0.854 (0.016)	5.5	0.22	3.45	0.16	0.58	0.62
Grand total	279	42	–	41	0.00157 (0.00006)	0.853 (0.016)	5.0	0.16	3.46	0.17	0.59	0.62

Breeding populations are Iceland, Svalbard, northern Nunavut, and southern Hudson Bay. Standard deviations are indicated in brackets where appropriate. *N*, sample size; *H*, number of haplotypes, Pr, number of private haplotypes, *S*, number of segregating sites. Nucleotide diversity (π), and haplotype diversity (*ĥ*) are shown with standard deviations in parentheses. *A*_T_, mean number of alleles; *P*_T_, mean number of private alleles; *A*_R_, mean number of alleles standardized to smallest population size (*n* = 5); *P*_R_, mean number of private alleles standardized to smallest population size (*n* = 5); *H*_O_, observed heterozygosity; *H*_E_, expected heterozygosity. Observed heterozygosity (*H*_O_) and expected heterozygosity (*H*_E_) are shown with standard deviations in parentheses.

Overall nucleotide diversity, π, was 0.00157 (Table 1). Nucleotide and haplotype diversity among breeding populations were lowest in Iceland (0.00063 and 0.660, respectively) and highest in southern Hudson Bay (0.00169 and 0.800, respectively). Nucleotide diversity and haplotype diversity in wintering populations were generally higher than in breeding populations (Table 1).

For microsatellites, deviations from linkage equilibrium were well below the expected number of false positives (22 expected, 10 observed), and none of the significant *p*‐values fell below the adjusted cutoff using sequential Bonferroni correction (see Table S4). Six locus‐population combinations showed deviations from Hardy–Weinberg equilibrium (see Table S5), which is slightly above the expected number of false positives for multiple calculations of *p*‐values at a .05 cutoff (five). Five of these were found in wintering populations, which are known to violate Hardy–Weinberg assumptions due to the presence of individuals from several distinct breeding populations, all with null allele frequencies ranging from 5% to 17%. One was found in Iceland at locus CM2668, with a null allele frequency of 15%. Null allele frequencies below 20% have limited effect on general population structure analyses (Chapuis & Estoup, 2007), and when analyses were run with or without locus, CM2668 results did not differ. Results are therefore reported here for all 10 loci.

Allelic diversity among the 10 microsatellite DNA loci ranged from 5 to 13 alleles, with a mean of 8.5 alleles/locus (Table S2). Samples from Svalbard showed higher observed heterozygosity than other breeding and wintering locations (Table 1). Allelic richness and expected heterozygosity were similar across all locations (Table 1).

### 3.2 Population Structure (Φ_ST_ and *F*_ST_)

Overall Φ_ST_ calculated for mtDNA across all breeding populations was 0.089 (*p *=* *.004). Overall *F*_ST_ calculated for microsatellites across breeding populations was 0.080 (*p *<* *.001). Both of these values were significantly higher than expected under panmixia (i.e., Φ_ST_ or *F*_ST_ = 0).

Pairwise Φ_ST_ and *F*_ST_ values, again calculated for mtDNA and microsatellites, respectively, indicate differentiation between most breeding populations, and between Iceland/Svalbard populations and most wintering locations (see Table 2). The exception was the Svalbard breeding population and Scotland, which had nonsignificant Φ_ST_ (*p *=* *.097) and *F*_ST_ (*p* = .999) values. Both Canadian breeding populations showed the opposite trend, with no measurable differentiation found between breeding population and wintering location. The exceptions to this were low but significant Φ_ST_ (Φ_ST_ = 0.093; *p* = .019) and *F*_ST_ (*F*_ST_ = 0.037; *p* < .001) values between northern Nunavut and Maine, and *F*_ST_ values between northern Nunavut and New Brunswick (*F*_ST_ = 0.026; *p* = .018). Finally, the Φ_ST_ value for Iceland and western Newfoundland was much higher (Φ_ST_ = 0.292; *p *=* *.034) than values found between Iceland and other North American wintering locations. In contrast, *F*_ST_ values between Iceland and western Newfoundland (*F*_ST_ = 0.061; *p *=* *.022) were much lower, though still different, than those between Iceland and eastern Newfoundland (*F*_ST_ = 0.146; *p *<* *.001).

**Table 2:** Pairwise estimates of Φ_ST_ and *F*_ST_ for breeding and wintering populations of Purple sandpipers (*Calidris maritima*)

	Maine	New Bruns.	Nova Scotia	Nfl	West Nfl	Scotland	Iceland	Svalbard	N. Nunavut	S. Hud. Bay
Maine	*	−0.007 (∞)	0.020 (24)	0.026 (19)	0.159 (3)	**0.087** **3** **(5)**	**0.136** **2** **(3)**	**0.180** **3** **(2)**	**0.093** **1** **(5)**	−0.088 (∞)
New Brunswick	0.002 (205)	*	−0.012 (∞)	−0.033 (∞)	0.044 (11)	0.037 (13)	**0.122** **2** **(4)**	**0.149** **2** **(3)**	0.048 (10)	−0.122 (∞)
Nova Scotia	−0.002 (∞)	−0.005 (∞)	*	0.005 (∞)	0.040 (12)	−0.002 (∞)	**0.069** **1** **(7)**	**0.069** **1** **(7)**	−0.012 (∞)	−0.076 (∞)
Newfoundland	**0.014** **1** **(36)**	0.001 (890)	−0.002 (∞)	*	−0.022 (∞)	0.024 (20)	**0.135** **2** **(3)**	**0.149** **2** **(3)**	0.038 (13)	−0.102 (∞)
Newfoundland‐W	0.013 (37)	0.001 (842)	0.017 (29)	0.015 (34)	*	−0.012 (∞)	**0.292** **1** **(1)**	0.102 (4)	0.008 (62)	0.107 (4)
Scotland	−0.038 (∞)	−0.052 (∞)	−0.051 (∞)	−0.070 (∞)	−0.074 (∞)	*	**0.055** **1** **(9)**	0.020 (24)	−0.020 (∞)	0.001 (423)
Iceland	**0.142** **3** **(3)**	**0.132** **3** **(3)**	**0.156** **3** **(3)**	**0.146** **3** **(3)**	**0.061** **1** **(8)**	**0.072** **3** **(6)**	*	**0.123** **2** **(4)**	**0.101** **2** **(4)**	0.173 (2)
Svalbard	**0.076** **3** **(6)**	**0.064** **3** **(7)**	**0.068** **3** **(7)**	**0.057** **3** **(8)**	**0.043** **1** **(11)**	−0.046 (∞)	**0.062** **3** **(8)**	*	0.017 (29)	**0.192** **1** **(2)**
N. Nunavut	**0.037** **3** **(13)**	**0.026** **1** **(19)**	0.016 (30)	−0.003 (∞)	0.055 (9)	−0.047 (∞)	**0.156** **3** **(3)**	**0.046** **2** **(10)**	*	0.018 (27)
S. Nunavut	−0.004 (∞)	−0.024 (∞)	−0.012 (∞)	0.002 (300)	−0.023 (∞)	−0.041 (∞)	**0.126** **3** **(3)**	**0.049** **2** **(10)**	0.013 (37)	*

Values of Φ_ST_ are above the diagonal, and *F*_ST_ values are below. Significant values (*p* < .05) are indicated in bold. Level of significance is indicated by superscript numbers, where ^1^ *p* < .05, ^2^ *p* < .01, and ^3^ *p* < .001. Number of migrants (mN) is given in brackets.

### 3.3 Phylogenetic analysis

**Figure 2:**
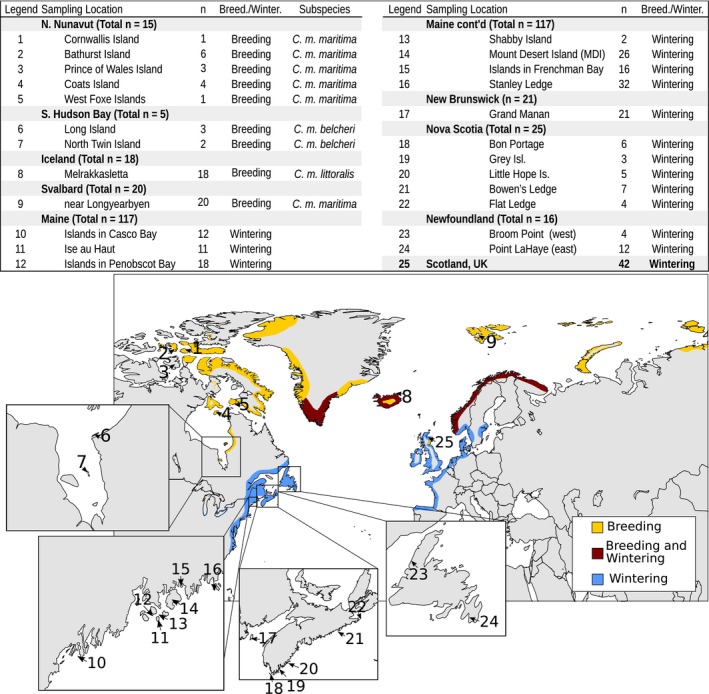
Range map of Purple Sandpipers, *Calidris maritima*. Numbers represent individual sampling locations. Bolded sites in the table represent sampling groups used in analysis. Shaded areas represent breeding (yellow), wintering (blue), and year‐round resident (red) populations. Map was redrawn from Payne and Pierce (2002) and the Global Register of Migratory Species (Riede, 2001)

The Bayesian (Figure 2), MLB, and maximum parsimony (not shown) trees all differed from each other in some branching patterns, reflecting the weak bootstrap and posterior probability (PP) support found for certain clades within each tree. The two largest clades were assigned the letters A and B for ease of discussion. Branch lengths were short throughout the tree, indicating recent divergence of haplotypes, no clade was monophyletic for a single breeding population (or putative subspecies), and no clade had posterior probabilities and bootstrap values above 95% and 60%, respectively, for any tree. All three trees displayed a “comb‐like” topology, and the lack of agreement among them reflects the general lack of resolution seen among haplotypes.

Clade A contained five haplotypes and was present in both the Bayesian and MLB trees. This clade has a PP of 96% and a bootstrap value of 52%. Clade B contained nine haplotypes and was found in all three trees. PP for this clade was 79%, and bootstrap values were 59% and 44% for MLB and MPBs, respectively. Notably, this clade was monophyletic for the two Canadian breeding populations of Purple Sandpipers (i.e., northern Nunavut and southern Hudson Bay), which may indicate that these haplotypes are unique to Canadian breeders. The remaining clades observed in these trees each contained only two haplotype groupings and had posterior probabilities or bootstrap values between 31% and 74%. The *p*‐distance between individuals within this clade and individuals outside the clade was .05 (or 0.5%).

Clade B was also present in the haplotype network. Haplotypes from all breeding locations were dispersed throughout most of the network outside Clade B, indicating limited geographic structure of Purple Sandpiper breeding populations. Branches were also quite short, with many singleton or low‐frequency haplotypes differing from a common haplotype by a single substitution.

**Figure 3:**
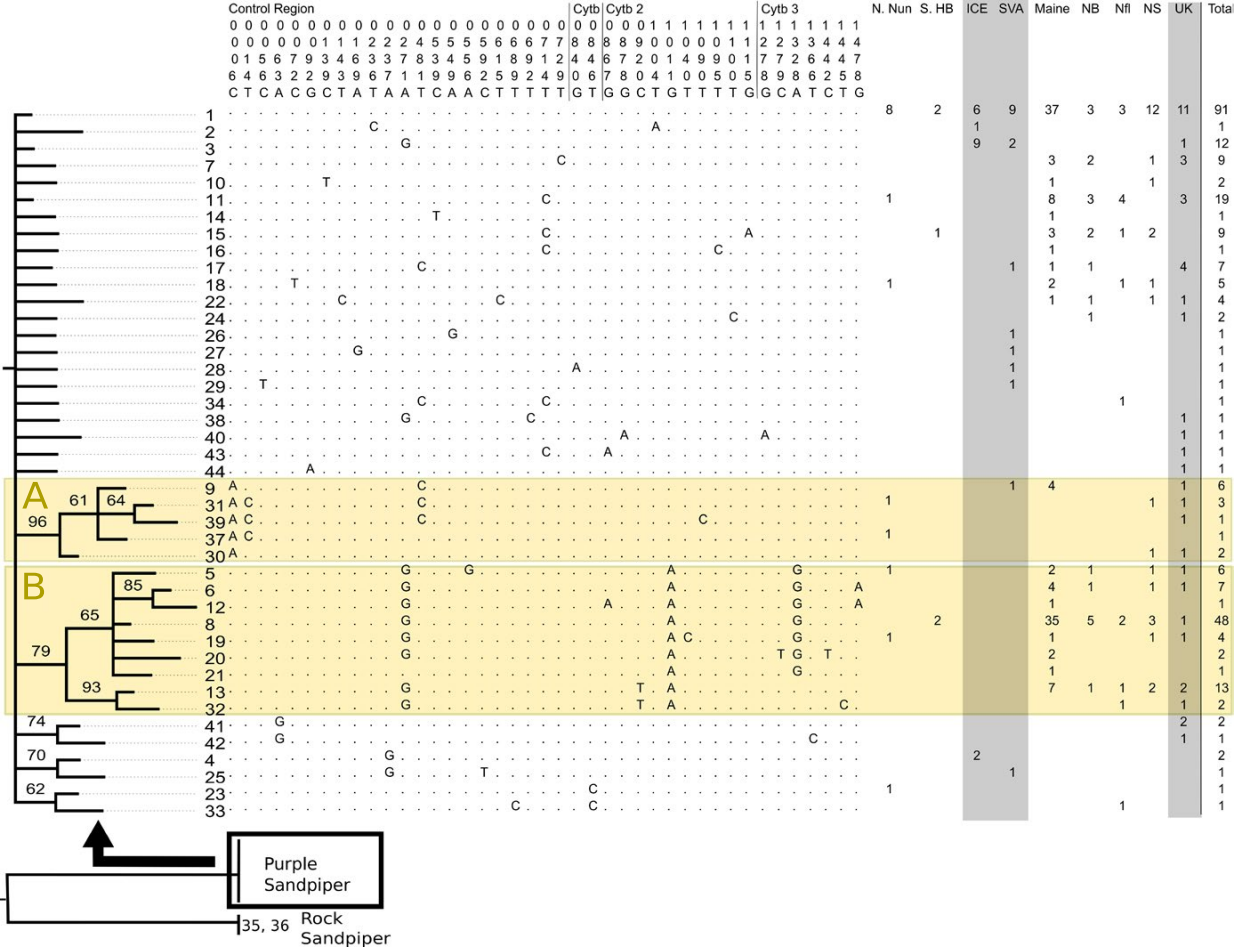
Rooted Bayesian phylogenetic tree (left), variable site matrix (center), and frequency of haplotypes across populations and in total (right) of Purple Sandpipers. Tree was constructed using HKY model of nucleotide evolution, with invariant sites, and a run length of 1,000,000. Site matrix shows variable positions relative to haplotype 1. Breeding populations and European wintering population frequencies are highlighted in gray. Tree was rooted using Rock Sandpiper DNA, shown at the bottom

Three circular connections were found in the haplotype network (Figure 3a). In an attempt to obtain additional sequence data that might help resolve the circular connections into clearly bifurcating relationships, a fragment of the ND2 mtDNA gene was amplified for one sample from each node involved in the circular network connections. However, all ND2 sequences examined were identical.

### 3.4 STRUCTURE clustering

**Figure 4:**
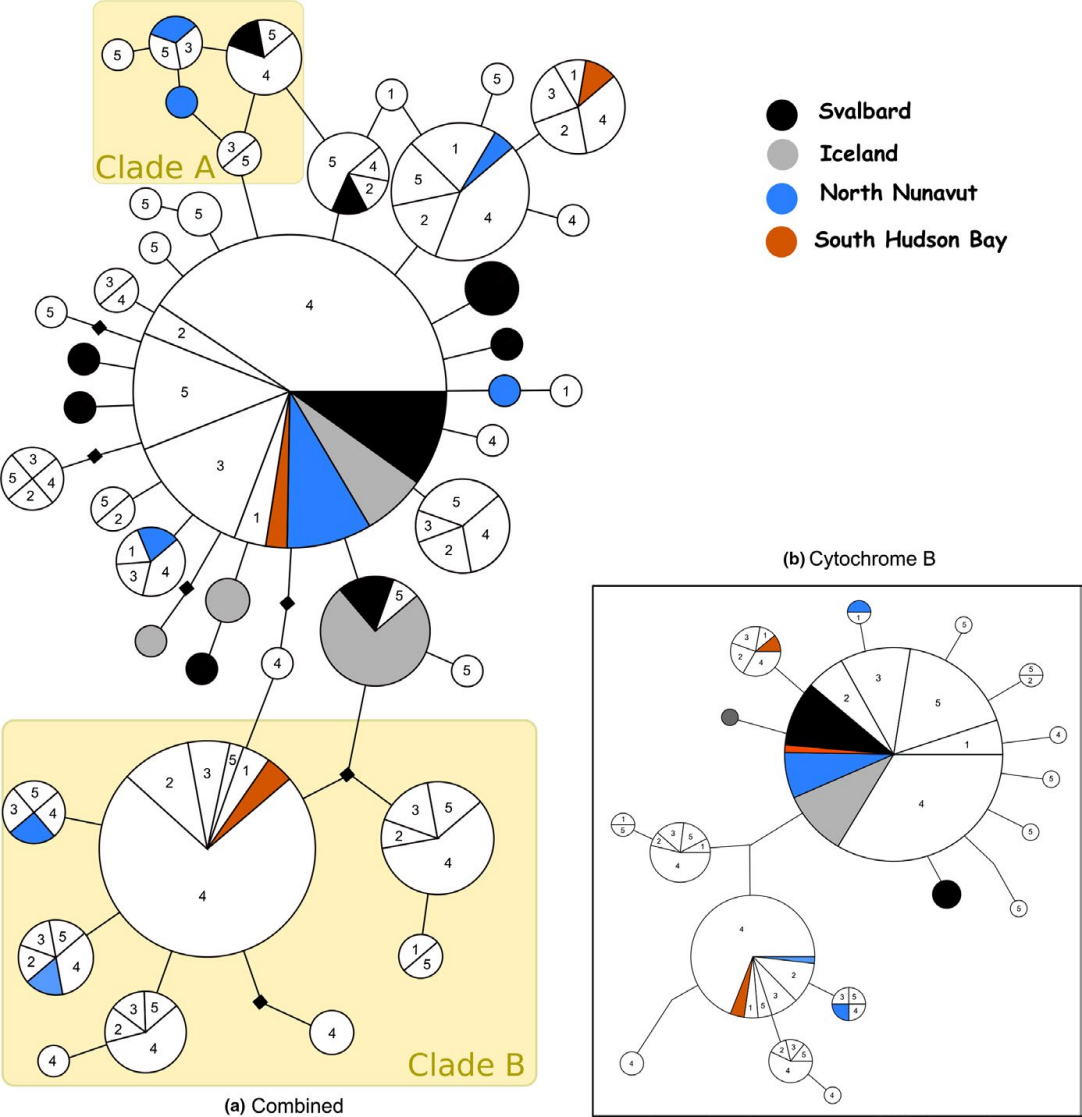
Unrooted statistical parsimony haplotype network of (a) combined/concatenated mitochondrial control region and cytochrome B fragments, totaling 1,534 base pairs and (b) cytochrome b fragments only, taken from 279 Purple Sandpipers. Nodes indicate individual haplotypes, and lines indicate 1‐base pair difference. Black squares indicate missing intermediate haplotypes. The size of a node roughly corresponds to the number of individuals with that haplotype, and the population origin of those individuals are marked by color or number. Wintering locations are shown in white and designated with numbers as follows: (1) Newfoundland, (2) New Brunswick, (3) Nova Scotia, (4) Maine, (5) Scotland. Breeding locations are shown in color, according to the legend

When all samples (breeding and wintering) were considered without a priori population information, the most likely number of clusters was four. However, only 43 individuals (15%) were assigned to a cluster with greater than or equal to 80% probability, and 127 (46%) birds were assigned to a cluster with 60% or greater probability. Iceland samples consistently showed highest assignment to one cluster, with Svalbard samples showing lower assignment to the same cluster (Figure 4). Southern Hudson Bay showed weak assignment to a second cluster, largely due to the two samples from North Twin Island near the southern end of Hudson Bay (see Figure 1) that were assigned to the second cluster with 71% and 91% probability. The remaining three southern Hudson Bay samples were from Long Island, which is farther north along the southeastern coast of Hudson Bay. The northern Nunavut samples showed assignment to a third cluster. The fourth cluster was not represented in the breeding samples apart from a single Nunavut breeder from Bathurst Island and may indicate an unsampled breeding population. Individuals in wintering populations showed moderate admixture and assignment to multiple clusters.

### 3.5 Demographic history

**Figure 5:**
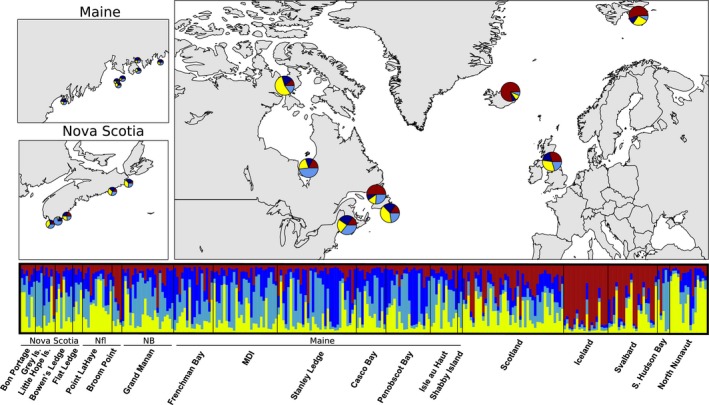
Below: Individual assignment of samples from breeding and wintering populations of Purple Sandpipers (*Calidris maritima*), to four genetic clusters calculated in STRUCTURE. Above: Overall assignment of each location to the four clusters

Mismatch distribution analysis of mtDNA sequences from all samples showed distributions that more closely matched that of a historically expanding population (Figure 5), although peaks were weakly bimodal rather than unimodal when all breeding populations were combined in a single analysis. In addition, the northern Nunavut and southern Hudson Bay populations showed deeper, multiple peaks, suggesting the presence of at least two historically distinct mtDNA lineages. When breeding populations were examined separately, Iceland and Svalbard samples both showed a unimodal distribution that more closely matched the expected curve of an expanding population. The northern Nunavut group showed a distinct bimodal distribution, while the southern Hudson Bay samples gave a ragged distribution. The raggedness statistic was significantly close to zero for northern Nunavut, as well as the group consisting of all samples pooled together.

**Figure 6:**
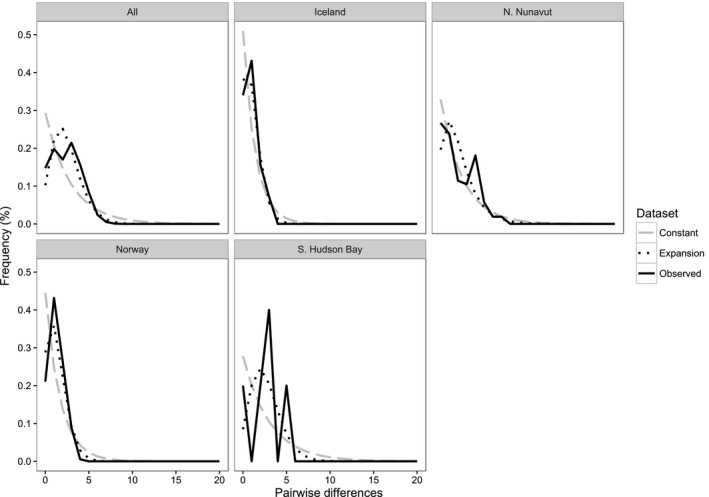
Mismatch distribution of concatenated mitochondrial control region and cytochrome b fragments, totaling 1,534 base pairs, of 279 Purple Sandpipers (*Calidris maritima*). Also shown are mismatch distributions of individual breeding populations. Observed frequencies of pairwise distances were plotted against expected frequencies given a neutral model of evolution and either population expansion or stable population size

Tajima's *D* was significantly lower than zero for the population as a whole (Table 3), as well as for Svalbard, indicating an excess of rare nucleotide substitutions compared to what is expected under a neutral model of evolution. Fu's *F*s was significantly lower than zero for Svalbard, northern Nunavut, and the population as a whole, indicating an excess of rare haplotypes. Ramos‐Onsin's *R* ^2^ was significantly close to zero in the population as a whole, Svalbard, and northern Nunavut, indicating an excess of singleton mutations compared to the overall number of nucleotide substitutions. Iceland and southern Hudson Bay showed no deviations from neutrality. Given the relative power of Fu's *F*s over Tajima's *D* (Fu, 1997; Ramos‐Onsins & Rozas, 2002) and *R* ^2^'s relative power over both at small samples sizes, the hypothesis of neutral evolution was rejected for Purple Sandpipers as a whole, as well as for the Svalbard and northern Nunavut breeding populations specifically.

**Table 3:** Demographic statistics for breeding populations of Purple Sandpipers (*Calidris maritima*), as well as all samples (breeding and wintering) pooled together. Numbers in bold represent tests statistically significant at *p*<.05

	Tajima's *D*	Fu's *F*s	*R* ^2^	*r*	τ
Iceland	−0.521 (.340)	−0.215 (.411)	.147 (.434)	.096 (.147)	0.961
Svalbard	−**1.748 (.013)**	−**5.531 (.000)**	**.072 (.000)**	.115 (.273)	1.249
N. Nunavut	−1.532 (.051)	−**3.010 (.020)**	**.072 (.000)**	**.039 (.041)**	0.849
S. Nunavut	0.562 (.695)	1.090 (.724)	.241 (.376)	.36 (.646)	2.023
Total	−**1.804 (.008)**	−**32.837 (.00)**	**.0288 (.036)**	**.0181 (.016)**	1.829

Tajima's *D* values, Fu's *F*s, Ramos‐Onsins and Rozas's *R* ^2^, raggedness index (*r*) and τ are represented with their statistical significance.

None of the Wilcoxon tests for heterozygosity excess was significant, and the mode shift test was significant only for southern Hudson Bay (data not shown). However, this population was below the minimum recommended number of samples (*n *=* *10), and thus, this result should be interpreted with caution.

## 4 Discussion

We found evidence of recent divergence in both mtDNA and nuDNA markers among four breeding populations of Purple Sandpipers, with samples from Iceland and Svalbard being the most distinctive for both data sets. We also found that wintering populations contained a mixture of genotypes and haplotypes, which is consistent with prior knowledge that wintering areas are populated by birds from multiple breeding grounds. There were notable differences in how closely the sampled breeding populations’ genotypes resembled different wintering populations, possibly reflecting migratory patterns. Mitochondrial markers assessed in this study showed deviations from neutrality, while microsatellites did not. Detailed interpretations of these patterns are discussed below.

### 4.1 Deviations from neutrality

Interpreting deviations from neutrality is challenging, as many demographic and selective processes may leave similar marks on an organism's genome (e.g., Li et al., 2012). The tests performed here that deviated from neutrality (i.e., Tajima's *D*, Fu's *F*, and Ramos‐Onsin's *R* ^2^) all showed a genetic signature that could reflect recent expansion of the Purple Sandpiper global breeding range, recovery from a strong bottleneck, or a recent selective sweep (Ramírez‐Soriano, Ramos‐Onsins, Rozas, Calafell, & Navarro, 2008). One method for determining whether a selective sweep took place is to examine data from multiple recombining loci (Galtier, Depaulis, & Barton, 2000). Demographic events such as bottlenecks and expansions should affect the entire genome, whereas genetic hitchhiking will generally affect only a small area (Galtier et al., 2000). The recent study of Purple Sandpipers by Barisas et al. (2015) examined multiple markers for both mtDNA and nuclear DNA. The mtDNA ND2 gene examined in that study showed a similar pattern to the combined control region and cytochrome b fragments here, with signs of expansion in Svalbard and northern Nunavut. For the nuclear markers examined by Barisas et al. (2015), the northern Nunavut population showed a significantly negative Tajima's *D* in the nuclear HMG‐2 marker; however, all other markers and populations did not show signs of deviation from neutrality suggesting that Purple Sandpiper mtDNA may have undergone purifying selection or a bottleneck. While mtDNA is often considered a neutral genetic marker, this is not always the case (Dowling, Friberg, & Lindell, 2008). Migratory birds with intense metabolic demands are known to have mitochondrial genomes that experience selective pressure (Toews, Mandic, Richards, & Irwin, 2014). Shorebirds that winter in northern climates generally have higher metabolic demands (Colwell, 2010), and this has been noted specifically for the closest relative to Purple Sandpipers, the Rock Sandpiper (Ruthrauff et al., 2015).

In the microsatellite data, there were no strong indications of a recent bottleneck in any of the Purple Sandpiper breeding populations, where “recent” is defined as after 0.25–2.5 times 2Ne generations ago (Cornuet & Luikart, 1996). Heterozygosity levels were moderate, and highest in Svalbard, as has been observed in other nuclear markers as well (Barisas et al., 2015), which may indicate that breeding populations in Iceland and Canada were established more recently than the Svalbard population.

### 4.2 Genetic structure in breeding populations

Measures of population divergence for mitochondrial DNA sequences and nuclear microsatellite alleles were largely in agreement. In contrast to the largely star‐like haplotype network, Φ_ST_, *F*_ST_, and STRUCTURE all found evidence of population structure among the breeding populations of Purple Sandpipers from Iceland, Svalbard, and Canada. STRUCTURE found evidence of some substructure between the southern Hudson Bay breeding population and northern Nunavut populations that was not reflected in pairwise Φ_ST_ or *F*_ST_ values. The two samples from North Twin Island seemed to drive most of this differentiation, indicating the possibility that the Purple Sandpipers in the south and southeastern part of Hudson Bay are not panmictic, but sample sizes are very small and this interpretation must be treated cautiously. In all tests, population differentiation among breeding populations appeared to follow a gradient in which Svalbard allelic frequencies were less differentiated from both Icelandic and Canadian populations than the Icelandic and Canadian populations were from each other.

A recent examination of the proposed subspecies of Purple Sandpipers (Barisas et al., 2015) found that when more breeding populations from the “intermediate” subspecies, *C. m. maritima*, were included in the morphological analysis, differences between subspecies failed to satisfy Amadon's rule for the designation of subspecies (Amadon, 1949). This rule specifies that 75% of individuals from a population be separable from 99% of overlapping populations (Amadon, 1949). Barisas et al. (2015) found evidence of multilocus genetic differentiation between Iceland, Svalbard, and northern Nunavut populations despite star‐like haplotype networks. These authors suggested that there was not enough evidence of genetic differentiation to support the designation of distinct subspecies. Barisas et al. (2015) also found greater differentiation between Iceland and Svalbard than between Iceland and northern Nunavut, a pattern that we observed in this study as well.

The pattern of pairwise Φ_ST_ and *F*_ST_ values observed between Iceland, Svalbard, and northern Nunavut breeding populations is similar to values found in a recent study of three Dunlin subspecies, *C. alpina schinzii*,* C. a. centralis*, and the intermediate subspecies *C. a. alpina* (Marthinsen, Wennerberg, & Lifjeld, 2007). However, in the case of Purple Sandpipers, the pattern of genetic relatedness among breeding locations does not correspond with putative subspecies (i.e., those proposed by Engelmoer & Roselaar, 1998). Rather, the pattern of population structuring demonstrated herein may indicate that Purple Sandpipers and Dunlin used the same glacial refugia. There is a distinct genetic lineage of Dunlin across much of northern Europe that includes western Greenland, Iceland, and Svalbard (and other Scandinavian countries and Russia), and another distinct lineage that includes much of Nunavut and parts of Hudson's Bay, Canada. This parallels the genetic differentiation between Canadian breeding birds and breeding birds from Iceland and Svalbard.

### 4.3 Genetic structure in wintering populations

Pairwise Φ_ST_ and *F*_ST_ between Iceland/Svalbard and most North American wintering locations were large and significant, with the exception of western Newfoundland. *F*_ST_ values between western Newfoundland and Iceland/Svalbard were much lower (though still significant) than values between Iceland/Svalbard and other North American wintering populations. These large differences suggest either that Purple Sandpipers from Iceland/Svalbard do not migrate to these areas of North America, or that they do so in relatively small numbers compared to migrants from northern Canada, Hudson Bay, the United Kingdom, and possibly unsampled areas such as Greenland. Purple Sandpipers from Iceland have been hypothesized to migrate to western Newfoundland in the winter, due to the large size of individuals in that particular region of Atlantic Canada (Hallgrimsson et al., 2012). The *F*_ST_ values reported here support a possible connection between the western Newfoundland wintering population and the Iceland breeding population (Hallgrimsson et al., 2012). In contrast, Φ_ST_ values between these same locations are much higher than between Iceland/Svalbard and other North American wintering locations. The four western Newfoundland samples in this study contained two mitochondrial haplotypes: One was the haplotype common to all sampled populations, and the other was a haplotype shared by some North American wintering birds but not with breeders from Iceland or Svalbard. Based on these data, we do not have any evidence that these particular western Newfoundland birds came from Iceland. In addition, *F*‐statistics for both mtDNA and nuDNA did not show a significant difference between western Newfoundland and other North American wintering populations. Nevertheless, microsatellite clustering for this population was noticeably different than other North American wintering populations. Additional sampling in the future will help further characterize the population of wintering migrants in western Newfoundland.

In addition to a possible migratory connection between western Newfoundland and Iceland/Svalbard, the low Φ_ST_ and *F*_ST_ values between Scotland and Canada/Svalbard populations support the recently described migration route between northern Canada and Scotland (Summers et al., 2014) and between Svalbard and northern Scotland (Hake et al., 1997; Summers et al., 2010). Summers et al. (2010) suggested that the Svalbard population contributes relatively few birds to the British wintering population. Rather, many wintering birds in eastern Britain are from southern Norway (Rae et al., 1986). Samples from these breeding populations will help determine whether breeding populations in southern Norway are genetically similar to the Svalbard population and thus how much the low values observed between Scotland and Svalbard reflect the presence of Svalbard and Norwegian breeders.

### 4.4 Diversity

While morphological variation can be an indicator of underlying genetic differentiation, many studies have found that subspecies defined by nonmolecular traits such as morphology and plumage often do not show corresponding genetic differentiation (e.g., Ball & Avise, 1992; Miller et al., 2013; Zink, 2004; Zink, Barrowclough, Atwood, & Blackwell‐Rago, 2000). This lack of congruence may be due to selective pressures on phenotypic traits that have caused them to diverge faster than neutral genetic markers (Haig et al., 2011).

The lack of genetic diversity within subspecies is especially common in birds that breed in the High Arctic, which typically display lower overall genetic variability, often attributed to recent bottlenecks during the Pleistocene glaciations (Piersma, 2003). Populations that have expanded after a population decline, as happens when recovering from a bottleneck, tend to display high haplotype diversity and low nucleotide diversity (Lounsberry et al., 2013). The very low nucleotide diversity values seen throughout the Purple Sandpiper range, but especially in breeding locations, do not seem to coincide with very low haplotype diversity, consistent with an interpretation that Purple Sandpiper populations have recently expanded. More data for mitochondrial‐encoded protein‐coding genes could help tease apart this interpretation from the alternative hypothesis of a selective sweep. Additional data would be needed to be able to examine the ratio of nonsynonymous to synonymous substitutions across a well‐resolved phylogeny (e.g., Hahn et al., 2002); much of our data is from the noncoding control region and our mitochondrial DNA phylogeny is largely unresolved.

Nucleotide diversity values for breeding locations roughly coincided with values obtained from the ND2 gene (Barisas et al., 2015). Overall, values for individual locations were less than a quarter of the observed nucleotide diversity observed in individual Dunlin lineages. Nucleotide diversity of all pooled Purple Sandpiper samples (0.00157) was two‐thirds of the lowest‐diversity Dunlin lineage (Alaskan; 0.0024) and three quarters of the average nucleotide diversity of Red Knots (*Calidris canutus*; Buehler, Baker, & Piersma, 2006). Knot diversity levels are theorized to have come from a single recent expansion from refugium after the Wisconsinan glaciation in North America (Buehler et al., 2006).

Overall, mtDNA nucleotide diversity was generally higher in Canadian populations than elsewhere, likely due to the presence of haplotypes from Clade B (Figure 2). This contrasts with patterns from the nuclear diversity measures presented here and in the recent study by Barisas et al. (2015) that consistently found higher diversity in Svalbard, possibly due to more extensive sampling in Canada in this study. Clade B could represent the presence of a separate mtDNA lineage in Canadian Purple Sandpipers, which was isolated in a second refugium during the last ice advance approximately 250,000 years ago. After Purple Sandpipers expanded outward from refugia, Canada was colonized by individuals from both lineages who then interbred. If this is the case, the genetic distance between the two mtDNA clusters is unusually low (compared to Dunlins: Buehler & Baker, 2005, Temmink's Stints, *Calidris temminckii*: Rönkä et al., 2012, and Common Eiders, *Somateria mollissima*: Sonsthagen, Talbot, Scribner, & Mccracken, 2011), but not unprecedented (e.g., Rock Sandpipers; Pruett & Winker, 2005). Both North American and Scotland wintering populations were represented in Clade B haplotypes, indicating that this clade is not limited to a particular migratory route. The general lack of structuring is even more pronounced than found in Rock Sandpipers by Pruett and Winker (2005), who showed that two of the four morphologically defined subspecies in *C. ptilocnemis* correspond to monophyletic mtDNA clades while two do not.

### 4.5 Management implications

The level and pattern of differentiation presented here, as well as in Barisas et al. (2015), suggest that the Iceland breeding population should be recognized as a distinct “management unit” (Moritz, 1994) on the basis of significant differentiation for at least one category of molecular marker (mitochondrial or nuclear). Indeed, because the Iceland population had significant Φ_ST_ and *F*_ST_ divergence values from all other locations (with the exception of Φ_ST_ from southern Hudson Bay), the Iceland population of Purple Sandpipers qualifies as an “evolutionarily significant unit” (ESU; Moritz, 1994), which represents some degree of reproductive isolation and possibly adaptive distinctiveness as well (Moritz, 2002). Because the southern Hudson Bay sample was so small, the lack of a significant difference between this population and the Iceland population as measured by Φ_ST_ should be discounted until further samples are analyzed from Nunavut. Somewhat less clear is what category should be applied to the Svalbard population and Canadian populations. Svalbard differed from Iceland and from the other breeding populations (with the exception of northern Nunavut) for both *F*_ST_ and Φ_ST_ (with the exception of northern Nunavut for Φ_ST_). It is not possible to discount the comparison between Svalbard and northern Nunavut given that the sample size was larger for northern Nunavut than southern Hudson Bay (as referred to above). We suggest that conservatively, Svalbard should be recognized as a separate MU while requiring further analysis to determine whether it should also be recognized as a distinct ESU. Similarly, the presence of a distinct mitochondrial lineage (i.e., Clade B; Figure 2) present in northern Nunavut and southern Hudson Bay breeding populations but not found in other breeding locations suggests that Canadian breeding birds should be recognized as a distinct management unit (Moritz, 1994; Topp & Winker, 2008).

In terms of genetic connectivity, the analyses presented here do not support a scenario in which Purple Sandpipers are considered to be a completely panmictic, homogeneous population. Limited gene flow among some breeding colonies may be due to breeding site fidelity (e.g., Payne & Pierce, 2002; Smith & Summers, 2005), which could contribute to levels of differentiation observed in this study. While there was some evidence for differentiation of Icelandic Purple Sandpipers, the presence of ancestral haplotypes shared between all populations prevents us from being able to definitively diagnose an Icelandic breeding origin for migratory birds. Wintering samples in North America indicate the presence of a genetic population not sampled in this study, possibly from Greenland or from an unsampled region of northern Canada. Two samples from Greenland were used in Barisas et al.'s (2015) review of putative subspecies in Purple Sandpipers, and they did not differ significantly from populations in Canada, Iceland, or Svalbard. However, due to the very low statistical power of such a small sample size, a larger number of individuals from this population needs to be examined to confirm this finding.

The gradual change in allele frequencies from Canadian populations to Svalbard to Iceland may indicate gene flow between Svalbard and the other two breeding populations. The majority of Svalbard breeders migrate to the Norwegian coast and western Sweden (Hake et al., 1997), but small numbers winter in northeast Scotland (Summers et al., 2010), mixing with a population of relatively long‐billed Purple Sandpipers from Canada (Summers et al., 2014). Gene flow between these two groups of wintering birds may account for the lower *F*_ST_ value between Svalbard and both Canadian breeding populations. A similar pattern of gene flow is found in birds that choose mates before migrating to their breeding grounds (e.g., Lesser Snow Geese *Chen caerulescens*; Quinn, 1992). Natal dispersal is extremely difficult to detect through banding; however, a shared wintering ground might facilitate the migration of naive birds to a different breeding population. The available evidence indicates that the Icelandic breeding population is resident (Summers et al., 1988) but in winter is joined by birds from other populations, presumably including birds from Canada. There is, however, a large difference in arrival time observed between morphologically defined groups in Scotland. Long‐billed birds from Canada arrive much later (October/November vs. July) and may depart later than the short‐billed Norwegian birds (Corse & Summers, 1999; Nicoll et al., 1988). This argues against wintering birds migrating to different breeding locations in the spring. Alternatively, the observed pattern of differentiation could be the result of incomplete lineage sorting due to recent expansion from a single refugium after the last glaciation. The high diversity values seen in nuclear markers here and in Barisas et al. (2015) may indicate a dispersal pattern of individuals radiating outward from Svalbard to Iceland and Canada. Further sampling of additional breeding populations (e.g., a large sample from Greenland) would shed more light on the genetic structure present in the rest of the range. The pattern of differentiation documented between breeding populations of Purple Sandpipers in this study and that by Barisas et al. (2015), however, is not consistent with the putative subspecies of Purple Sandpipers identified by Engelmoer and Roselaar (1998). Large‐scale next‐generation single nucleotide polymorphism studies could provide for greater discrimination between breeding populations, both due to the large number of markers throughout the genome and the inclusion of markers that have undergone selection due to local adaptation (Freamo, O'Reilly, Berg, Lien, & Boulding, 2011).

Many wintering populations of shorebirds in North America have shown evidence of decline in recent years (Andres et al., 2012; Austin, Collier, & Rehfisch, 2008; Corse & Summers, 2009; Rehfisch, Holloway, & Austin, 2003; Summers, 2014). This is particularly evident along the Atlantic coast of Canada and the northeastern United States, where a recent survey showed that 22 of 30 migrating shorebird species were declining (Bart, Brown, Harrington, & Morrison, 2007). While population trends are poorly known for Purple Sandpipers, wildlife managers suspect that the species is in decline (Gratto‐Trevor et al., 2011). Robust estimates of population numbers and trends have been hampered by the difficulty of counting birds in remote breeding habitats and at nonbreeding sites (Payne & Pierce, 2002). A recent study by Mallory et al. (2016) found that Purple Sandpipers wintering in Nova Scotia consumed principally marine invertebrates, dominated by periwinkles (*Littorina* spp.) and mussels (*Mytilus* spp.), as well as other arthropods, a diet similar to those studied in Europe. Wintering Purple Sandpipers rely heavily on habitat containing intertidal red seaweed *(Mastocarpus stellatu*s), and to a lesser extent on Rockweed *(Ascophyllum nodosum*), and commercial harvesting of marine alga (Seeley & Schlesinger, 2012) could contribute to local declines. The effects of invading green crabs (*Carcinus maenus*) in Atlantic Canada could also negatively affect wintering populations, and consequently breeding populations from Canadian and European locations that winter here, by depleting mussels and periwinkle populations, both of which have been shown to be favored prey of this crab (Singh, 1991). As noted by Colwell (2010), wintering shorebirds must deal with extreme challenges of cool, wet, and windy conditions while feeding and roosting in northern estuaries. Any additional challenges as a consequence of anthropogenic activities or invading species could significantly tip the balance against their survival during this crucial phase of their life cycle.

At all stages of its life cycle, the Purple Sandpiper inhabits remote, often inhospitable habitats (Payne & Pierce, 2002), and thus it has been a challenging species on which to gather sound information regarding population size and trends. New technologies such as small telemetry units (e.g., Summers et al., 2014) are providing new insights into movements among breeding populations, and our genetic analyses have provided additional insights into the broader population structure of the species. Despite these efforts, this little bird retains many secrets, particularly regarding its phylogeography, which are key to ensuring sustainable management of breeding populations. Future conservation efforts regarding Purple Sandpipers will clearly require international collaboration to help monitor and maintain populations of this species across their global range, and in particular, we stress the need for genetic sampling of birds in those areas underrepresented in this and earlier studies, notably from West Greenland and the western Canadian Arctic.

## Conflict of Interest

None declared.

## Author Contributions

Sample acquisition was coordinated by Mark Mallory, and samples were also contributed by Mark Elderkin, Glenn Mittelhauser, Snaebjörn Pálsson, Julie Paquet, Lindsay Tudor, and Ron Summers. Project funding was obtained by Mark Mallory, Steve Mockford, Greg Robertson, Don Stewart, and others, and reagents and laboratory space were provided by Don Stewart. Data were obtained and analyzed by Nathalie LeBlanc, with advice from Don Stewart and Mark Mallory. Paper was drafted by Nathalie LeBlanc, and edited by all coauthors.

## Data Accessibility

Control Region and Cytochrome B sequences for Purple Sandpipers and Rock Sandpipers have been submitted to GenBank under accession numbers KX793138–KX793697. Microsatellite alleles can be found in the Dryad repository (DOI: doi:10.5061/dryad.tn1h7).
