# Supplementary material for: Population structure of Purple Sandpipers (Calidris maritima) as revealed by mitochondrial DNA and microsatellites
Source: Ecol Evol. 2017 Mar 31;7(9):3225–42. doi: 10.1002/ece3.2927 (PMC5415539; doi:10.1002/ece3.2927)
Supplement: Supplementary file 1 [file ECE3-7-3225-s001.pdf]

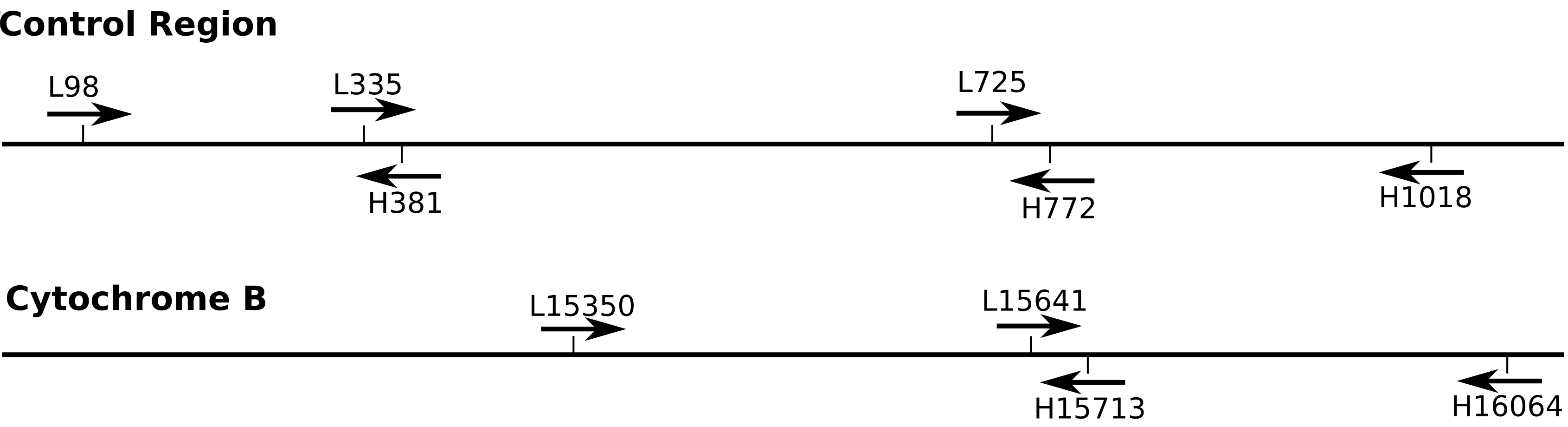
Control Region
L98
L335
H381
L725
H772
H1018
Cytochrome B
L15350
L15641
H15713
H16064
