# Supplementary material for: Population structure of Purple Sandpipers (Calidris maritima) as revealed by mitochondrial DNA and microsatellites
Source: Ecol Evol. 2017 Mar 31;7(9):3225–42. doi: 10.1002/ece3.2927 (PMC5415539; doi:10.1002/ece3.2927)
Supplement: Supplementary file 2 [file ECE3-7-3225-s002.doc]

**Table S1.** List of primers used to amplify and sequence mtDNA fragments of the control-region and cytochrome b of purple sandpipers (*Calidris maritima*). Primers are numbered according to their approximate location in the mtDNA genome. Primers marked with an asterisk were used to amplify Rock Sandpiper (*Calidris ptilocnemis*) samples.

________________________________________________________________________

Primer Name Sequence (5' – 3') Source

Control Region Primers

L98* GCATGTAATTTGGGCATTTTTTG Wenink et al. 1993

H381 AACCTGGTACGACTGGTGTG In Lab^1^

L335 ACAGCTCGGAAACTCTCGAA In Lab^1^

H772* AAACACTTGAAACCGTCTCAT Wenink et al. 1993

L725* GCCCTCAGGCGTTACTGA In Lab^1^

H1018 GTTCATCTATTCGTTTATGGTT In Lab^1^

H1030* CGAATAGATGAACGCAAACG In Lab^1^

Cytochrome B Primers

L15350* TTACAAACCTATTCTCAGG Pruett and Winker 2005

H15713 TGGGGAGGTGTGACTAGAGG In Lab^1^

L15641 ACCCCAGCAAACCCTCTAGT In Lab^1^

H16064* CTTCAGTTTTTGGTTTACAAGACC Pruett and Winker 2005

^1^ Primers were developed in-lab using the software Primer 3.

**Table S2.** Characteristics and sequence of 10 microsatellite loci developed for purple sandpipers (*Calidris maritima*).

Locus Sequence (5' – 3') Repeat Motif Size (bp) N N_A_ H_o_ H_E_ A_R_ T_A_

CM2668 F: TACCAGGCTTCTTCTCGGTC (CA) _14_ 146 - 170^1^ 264 11 0.46 0.49 3.02 56

R: CTCTTGGCGAGGAGGTAAGG

CM2988 F: TGCTAGATCATTGGGAAAGCAG (AC) _12_ 228 – 242 268 7 0.57 0.62 3.62 56

R: ATCTGCAGTCCGGAAAGTGG

CM0705 F: CAGAGGTGCCACTCCAAAAC (AC) _12_ 139 – 155^1^ 233 6 0.60 0.60 2.87 56

R: TGCTGTAGTCAGGTGAGAGG

CM0997 F: CCTGGTGGGCATGAACAATC (AC) _18_ 227 - 247 258 10 0.62 0.71 3.86 56

R: AGCAGACCTGTACACTGACG

CM0296 F: GCTTGTTCAAGAGCTGGTGG (CA) _11_ 229 – 251 264 9 0.69 0.76 4.19 56

R: TGGGCTCCTGTTCCATTACC

CM3007 F: AATTCTCTGTGAGCGCGTTG (GT) _11_ 88 – 100^1^ 270 5 0.39 0.49 2.17 56

R: TAGTCCTGGCTGTTGACCTG

CM1669 F: TTGGAAACAACGCCTGTAGC (AC) _14_ 232 - 246 251 7 0.49 0.63 3.16 56

R: ATTTGCATGGCCTGTTAGCC

CM2198 F: GCCCCAGTTCTCAATGTGTC (AC) _19_ 91 – 117^1^ 253 9 0.70 0.71 4.37 56

R: CTAGAAGGTCGAGCAGGAGG

CM1422 F: CTGTTAGTTGCCAGGGGGAG (GT) _16_ 71 – 97 272 13 0.79 0.78 4.87 56

R: GTCAAATACCTGTGGTGCCAG

CM3547 F: GACAGACAACCTAAGTAGGCATC (AC) _13_ 72 – 90 265 8 0.55 0.58 3.83 56

R: CATTTAAGGCGCGCTCTGTG

N = number of individuals genotyped to obtain the data to the right, N_A_ = number of alleles, Ho = observed heterozygosity, H_E_ = expected heterozygosity, A_R_ = mean number of alleles standardized to a population size of 5, and T_A_ = annealing temperature.

^1^ The ‘smaller’ fragment in its duplex PCR reaction.

Figure S1. Primer Schematic - mtDNA
